# Proposal of Takagi–Sugeno Fuzzy-PI Controller Hardware

**DOI:** 10.3390/s20071996

**Published:** 2020-04-02

**Authors:** Sérgio N. Silva, Felipe F. Lopes, Carlos Valderrama, Marcelo A. C. Fernandes

**Affiliations:** 1 Laboratory of Machine Learning and Intelligent Instrumentation, IMD/nPITI, Federal University of Rio Grande do Norte, Natal 59078-970, Brazil; sergionatan@dca.ufrn.br (S.N.S.); lopesffernandes@gmail.com (F.F.L.); 2 Department of Electronics & Microelectronics, Polytechnic Faculty, University of Mons, Mons 7000, Belgium; CarlosAlberto.VALDERRAMASAKUYAMA@umons.ac.be; 3 Department of Computer and Automation Engineering, Federal University of Rio Grande do Norte, Natal 59078-970, Brazil

**Keywords:** FPGA, hardware, Takagi–Sugeno, fuzzy, fuzzy-PI

## Abstract

This work proposes dedicated hardware for an intelligent control system on Field Programmable Gate Array (FPGA). The intelligent system is represented as Takagi–Sugeno Fuzzy-PI controller. The implementation uses a fully parallel strategy associated with a hybrid bit format scheme (fixed-point and floating-point). Two hardware designs are proposed; the first one uses a single clock cycle processing architecture, and the other uses a pipeline scheme. The bit accuracy was tested by simulation with a nonlinear control system of a robotic manipulator. The area, throughput, and dynamic power consumption of the implemented hardware are used to validate and compare the results of this proposal. The results achieved allow the use of the proposed hardware in applications with high-throughput, low-power and ultra-low-latency requirements such as teleoperation of robot manipulators, tactile internet, or industry 4.0 automation, among others.

## 1. Introduction

Systems based on Fuzzy Logic (FL), have been used in many industrial and commercial applications such as robotics, automation, control, and classification problems. Unlike high data volume systems, such as Big Data and Mining of Massive Datasets (MMD) [1,2,3], one of the great advantages of Fuzzy Logic is its ability to work with incomplete or inaccurate information.

Intelligent systems based on production rules that use Fuzzy Logic in the inference process are called in the literature Fuzzy Systems (FS) [4]. Among the existing inference strategies, the most used, the Mamdani and the Takagi–Sugeno, are differentiated by the final stage of the inference process [5,6,7,8,9,10,11,12,13,14,15,16,17,18,19,20].

The interest in the development of dedicated hardware implementing Fuzzy Systems has increased due to the demand for high-throughput, low-power, and ultra-low-latency control systems for emerging applications such as the tactile Internet [21,22], the Internet of Things (IoT), and Industry 4.0, where the problems associated with processing, power, latency, and miniaturization are fundamental. Robotic manipulators used on the tactile internet need a high-throughput and ultra-low-latency control system, and this can be achieved with dedicated hardware [21].

The development of dedicated hardware, in addition to speeding up parallel processing, makes it possible to operate with clocks adapted to low-power consumption [23,24,25,26,27,28,29]. The works presented in [30,31,32,33,34,35,36,37] propose implementations of FS on reconfigurable hardware (Field Programmable Gate Array—FPGA), showing the possibilities associated with the acceleration of fuzzy inference processes having a high degree of parallelization. Other works propose specific implementations of Fuzzy Control Systems (FCS) using the Fuzzy Mamdani Inference Machine (M-FIM) and the Takagi–Sugeno Fuzzy Inference Machine (TS-FIM) [5,6,7,8,9,10,11,12,13,14,15,16,17,18,19,20]. The works presented in [38,39,40] propose the Takagi–Sugeno hardware acceleration for other types of application fields.

This work aims to develop a new hardware proposal for a Fuzzy-PI controller with TS-FIM. Unlike most of the works presented, this project offers a fully parallel scheme associated with a hybrid platform using fixed-point and floating-point representations. Two TS-FIM hardware modules have been proposed, the first (here called TS-FIM module one-shot) takes one sample time to execute the TS-FIM, and the second (called here the TS-FIM module pipeline) uses registers inside the TS-FIM. Two pieces of Fuzzy-PI controller hardware have been proposed, one for the TS-FIM one-shot module and another for the TS-FIM module pipeline. The proposed hardware has been implemented, tested, and validated on a Xilinx Virtex 6 FPGA (6-bit LUTs) (San Jose, CA, USA). The synthesis results, in terms of size, resources, and throughput, are presented according to the number of bits and the type of numerical precision. Already, the physical area on the target FPGA reaches less than 7%. The implementation achieved a throughput between 10 and 18Msps (Mega samples per second), and between 490 and 882Mflips (Mega fuzzy logic inferences per second). Validation results on a feedback control system are also presented, in which satisfactory performance has been obtained for a small number of representation bits. Comparisons of results with other proposals in the literature in terms of throughput, hardware resources, and dynamic power savings will also be presented.

## 2. Related Works

In [30], a high-performance FPGA Mamdani fuzzy processor is presented. The processor achieved a throughput of about 5Mflips at a clock frequency of about 40MHz, and it was designed for 256 rules and 16 inputs with 16 bits. The proposal used a semi-parallel implementation and thus reduced the number of the operations per Hz. The work presented in [30] has about $\frac{5}{40}=0.125\;\mathrm{flips}/\mathrm{Hz}$ and the work proposed here can achieve about $\frac{256*40}{40}=256\;\mathrm{flips}/\mathrm{Hz}$ due to the fully parallel hardware scheme used. The significant difference between throughput and operation frequency also implies a high power consumption [41]. The work presented in [31] uses a Mamdani inference machine and the throughput in Mflips is about 48.23Mflips. The hardware was designed to operate with eight bits, four inputs, nine rules, and one output. Similar to the work presented in [30], the proposal introduced in [31] adopted a semi-parallel implementation, and this way decreased the throughput and increased power consumption. Other Mamdani implementations following the same strategy are also found in [32,33,34,35].

A multivariate Takagi–Sugeno fuzzy controller on FPGA is proposed in [5]. The hardware is applied to the temperature and humidity controller for a chicken incubator, and it was projected to two inputs, six rules, and three outputs. When compared to other works, the hardware proposed in [5] achieved a low throughput of about 6Mflips. A hardware accelerator architecture for a Takagi–Sugeno fuzzy controller is proposed in [7], and this proposal achieved a throughput about 1.56Msps with three inputs, two outputs, and 24 bits.

In [11,12,13], a design methodology for rapid development of fuzzy controllers on FPGAs was developed. For the case with two inputs, 35 rules and one output (vehicle parking problem), the proposed hardware achieved a maximum clock of about 66.251MHz with 10 bits. However, the TS-FIM takes 10 clocks to complete the inference step, and this decreases the throughput, and it increases the power consumption.

The implementation presented in [14] aims at creating a hardware scheme of a fuzzy logic controller on FPGA for the maximum power point tracking in photo-voltaic systems. The implementation takes six clock cycles over 10MHz, and this is equivalent to a throughput of about $\frac{10\;\mathrm{MHz}}{6}\approx 1.67\;\mathrm{Msps}$. In [16], a Mamdani fuzzy logic controller on FPGA was proposed. The hardware carries out a throughput of about 25Mflips with two inputs, 49 rules.

The work presented in [17] implements a semi-parallel digital fuzzy logic controller on FPGA. The work achieved about 16Msps per clock frequency of 200MHz, that is, 0.08Msps/MHz. On the other hand, this manuscript uses a fully parallel approach, and it achieves 1Msps/MHz; in other words, it can execute more operations per clock cycle. In the same direction, the proposals presented in [18,20] shows a semi-parallel fuzzy control hardware with low-throughput, about 1Msp.

Thus, this manuscript proposes a hardware architecture for the Fuzzy-PI control system. Unlike the works presented in the literature, the strategy proposed here uses a fully parallel scheme associated with a hybrid use in the bit format (fixed and floating-point). After several comparisons with other implementations of the literature, the scheme proposed here showed significant gains in processing speed (throughput) and dynamic power savings.

## 3. Takagi–Sugeno Fuzzy-PI Controller

**Figure 1:**
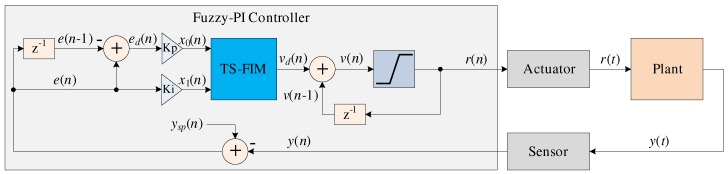
Architecture of the Fuzzy-PI feedback control system operating a generic plant.

Figure 1 shows the Fuzzy-PI intelligent control system operating a generic plant [4,42,43]. The plant output variable y(t) is called the controlled variable (or controlled signal), and it can admit several kinds of physical measurements such as level, angular velocity, linear velocity, angle, and others depending on the plant characteristics. The controlled variable, y(t), passes through a sensor that converts the physical measure into a proportional electrical signal that is discretized at a sampling rate, $t_{s}$, generating the signal, y(n).

The plant drives the kind of sensor that will be used. For level control in tanks used in industrial automation, the sensor can be characterized by the pressure sensor. For robotics applications (manipulators or mobile robotics), the sensor can be a position (capture angle information) or an encoder sensor (capture angular or linear velocity information).

In the *n*-th time, the Fuzzy-PI controller (see Figure 1) uses the signal, y(n), and it calculates the error signal, e(n), and difference of error, $e_{d}\left(n\right)$. The signal e(n) is expressed by

(1) $$e\left(n\right)=y_{sp}\left(n\right)-y\left(n\right),$$

where the $y_{sp}\left(n\right)$ is the reference signal, also called the set point variable, and the signal $e_{d}\left(n\right)$ is expressed by

(2) $$e_{d}\left(n\right)=e\left(n\right)-e\left(n-1\right).$$

After the computation of the signals e(n) and $e_{d}\left(n\right)$, the Fuzzy-PI controller generate the signals $x_{1}\left(n\right)$ and $x_{2}\left(n\right)$, which can be expressed as

(3) $$x_{0}\left(n\right)=\mathrm{Kp}\times e_{d}\left(n\right)$$

and

(4) $$x_{1}\left(n\right)=\mathrm{Ki}\times e\left(n\right).$$

The variables Kp and Ki represent the proportional and the integration gains, respectively [4,42,43]. Subsequently, the signals $x_{0}\left(n\right)$ and $x_{1}\left(n\right)$ are sent to the Takagi-Sugeno fuzzy inference; called in this article Takagi-Sugeno - Fuzzy Inference Machine or TS-FIM (see Figure 1).

The TS-FIM is formed by three stages called fuzzification, operation of the rules (or rules evaluation), and defuzzification (the output function) [4]. In the fuzzification, each *i*-th input signal $x_{i}\left(n\right)$ is applied to a set of $F_{i}$ pertinence functions whose output can be expressed as

(5) $$f_{i,j}\left(n\right)=\mu_{i,j}\left(x_{i}\left(n\right)\right)\;\mathrm{for}\;j=0,\ldots ,F_{i},$$

where $\mu_{i,j}\left(\cdot \right)$ is the *j*-th membership function of the *i*-th input and $f_{i,j}\left(n\right)$ is the output of the fuzzification step associated with the *j*-th membership function and the *i*-th input in the *n*-th time.

For two inputs, $x_{0}\left(n\right)$ and $x_{1}\left(n\right)$, the TS-FIM generates a set of $F_{0}+F_{1}$ fuzzy signals ($f_{0,j}$ and $f_{1,j}$) and these signals are processed by a set of $F_{0}F_{1}$ rules in the rules evaluation step. Each *g*-th rule can be expressed as

(6) $$o_{g}=\min\left(f_{0,l},f_{1,k}\right)\;\mathrm{for}\;g=0,\ldots ,\left(F_{0}F_{1}\right)-1,$$

where $g=F_{0,l+k}$ for $\left(l,k\right)=\left(0,0\right),\left(0,1\right),\ldots ,\left(F_{0}-1,F_{1}-1\right)$. Finally, the output (defuzzification) of TS-FIM, called here $v_{d}\left(n\right)$, can be expressed as

(7) $$v_{d}\left(n\right)=\frac{a(n)}{b(n)}=\frac{\sum_{g=1}^{\left(F_{0}F_{1}\right)-1}a_{g}}{\sum_{g=0}^{\left(F_{0}F_{1}\right)-1}o_{g}}=\frac{\sum_{g=1}^{\left(F_{0}F_{1}\right)-1}o_{g}\times \left(A_{g}x_{0}\left(n\right)+B_{g}x_{1}\left(n\right)+C_{g}\right)}{\sum_{g=0}^{\left(F_{0}F_{1}\right)-1}o_{g}},$$

where $A_{g}$, $B_{g}$, and $C_{g}$ are parameters defined during the project [4]. Thus, it can be said that every *n*-th instant TS-FIM receives as input $x_{0}\left(n\right)$ and $x_{1}\left(n\right)$ and generates as output v(n), that is,

(8) $$v_{d}\left(n\right)=TSFIM\left(x_{0}\left(n\right),x_{1}\left(n\right)\right),$$

where $TSFIM\left(\cdot \right)$ is a function that represents TS-FIM.

After the TS-FIM processing, the Fuzzy-PI controller integrates the signal $v_{d}\left(n\right)$ generating the signal v(n) (see Figure 1). The signal $v_{d}\left(n\right)$ is the output of the Fuzzy-PI controller, and it can be expressed as

(9) $$v\left(n\right)=v_{d}\left(n\right)+v\left(n-1\right).$$

This signal saturates beyond $v_{\min}$ and $v_{\max}$, generating the signal r(n) that it is expressed as

(10) $$r\left(n\right)=\left\{\begin{array}{cc} v_{\max} & \;\mathrm{for}\;v\left(n\right)>v_{\max}\\ v(n)\\ v_{\min} & \;\mathrm{for}\;v\left(n\right)<v_{\min} \end{array}\right..$$

Finally, the signal r(n) is sent to an actuator, which transforms the discrete signal into a continuous signal, r(t), to be applied to the plant.

## 4. Hardware Proposal

**Figure 2:**
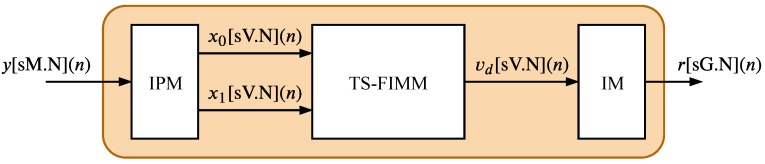
Overview of Fuzzy-PI controller proposed architecture.

The general structure of the proposed hardware implementation, composed of three main modules called input processing (IPM), TS-FIM (TS-FIMM) and integration (IM), is represented in Figure 2. The hardware was developed for the most part using a fixed-point format for the variables, in which, for any given variable, the notations [uT.W] and [sT.W] indicate that the variable is formed by T bits of which W are intended for the fractional part and the symbols “u” and “s” indicate that the variable is signed or unsigned, respectively. For signed variables, the number of bits intended for the integer part is T−W−1 and, for unsigned variables, the number of bits for the integer part is T−W.

### 4.1. Input Processing Module (IPM)

**Figure 3:**
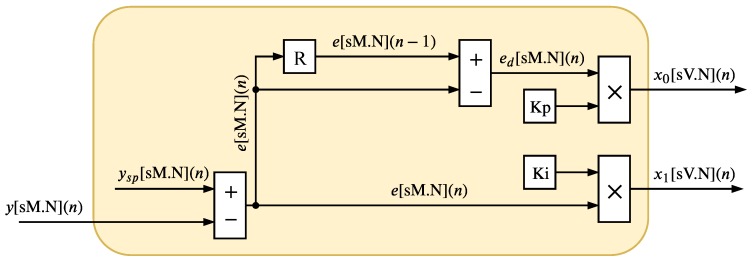
Hardware architecture of IPM.

The IPM (shown in Figure 3) is responsible for processing the control signal sent by the plant to the input of the Fuzzy-PI controller. The IPM computes the Equations (1)–(4). The signals associated with this module were implemented with *M* bits, where one is reserved for the sign and *N* for the fractional part, so the value of *M* can be expressed as

(11) $$M=N+\log_{2}\left(\left\lceil y_{max}\right\rceil \right)+1,$$

where $y_{max}$ represents the maximum value, in modulus, of the process variable, y(n).

The constant parameters Kp and Ki of the gain modules (see Equations (3) and (4)) must be designed to maintain the output signals $x_{0}\left[V.N\right]\left(n\right)$ and $x_{1}\left[V.N\right]\left(n\right)$ between −1 and 1. However, it is important to note that the two gain modules can saturate the signal in [V.N](n) bits after the multiplication.

### 4.2. TS-FIM Module (TS-FIMM)

The TS-FIMM is composed of three hardware components: Membership Function Module (MFM), Operation Module (OM), and Output Function Module (OFM). The MFM is the first module associated with TS-FIMM, and it corresponds to the fuzzification process, the OM component completes the rules evaluation phase and the OFM performs the defuzzification step (see Section 3). This work proposes two alternative architectures for the TS-FIMM.

**Figure 4:**
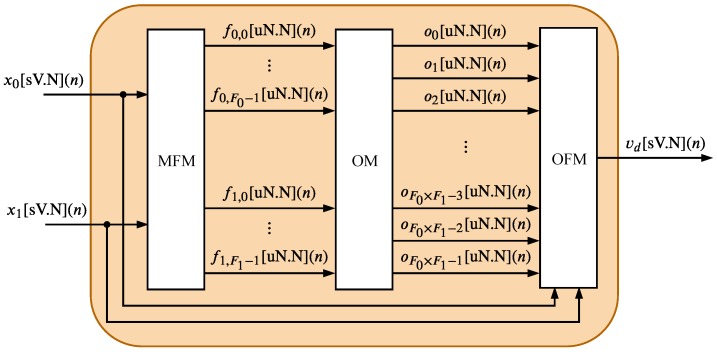
Hardware architecture of TS-FIMM One-Shot (TS-FIMM-OS).

**Figure 5:**
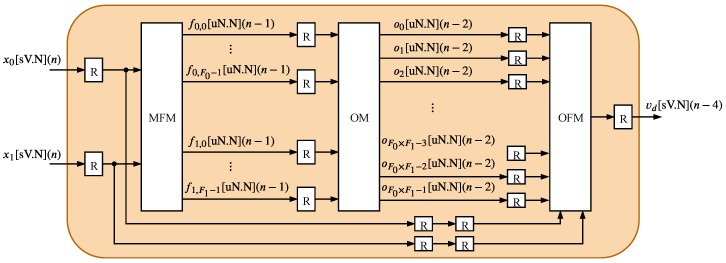
Hardware architecture of TS-FIMM Pipeline (TS-FIMM-P).

The first proposed architecture, presented in Figure 4 and called here TS-FIMM One-Shot (TS-FIMM-OS), performs all modules MFM, OM, and OFM in one sampling time, in other words, it takes a time interval between samples to generate the *n*-th output associated with the *n*-th input. The second one, presented in Figure 5 and called here TS-FIMM Pipeline (TS-FIMM-P), uses registers (blocks called R in Figure 4) among the input, MFM, OM, OFM, and output. The TS-FIMM-P has a latency of four sampling times to perform all modules MFM, OM, and OFM, in other words, there is a delay of four samples between the *n*-th output and *n*-th input.

The TS-FIMM-OS will finally have a longer execution time than TS-FIMM-P because it has a longer critical path; however, the TS-FIMM-OS does not have a delay. It is important to empathize that the delay inside the feedback control can take a system to instability. Indeed, the instability degree depends on the system and how long the delay is. The instability will depend on the characteristics of the system and the size of the delay [44]. On the other hand, the pipeline scheme associated with the TS-FIMM-P has a shorter critical path, which allows a higher throughput compared to the TS-FIMM-OS.

#### 4.2.1. Membership Function Module (MFM)

**Figure 6:**
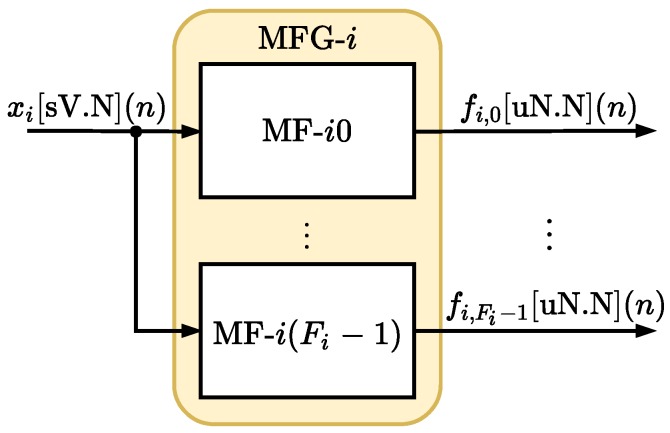
Hardware architecture of module MFG-*i* associated with the *i*-th input, $x_{i}\left[\mathrm{sV}.N\right]\left(n\right)$.

In the MFM, each *i*-th input variable is associated with a module that collects $F_{i}$ membership functions, called here the Membership Function Group (MFG). Figure 6 shows the *i*-th MFG, or MFG-i, related to the *i*-th input $x_{i}\left[\mathrm{sV}.N\right]\left(n\right)$.

Each MFG-i collects $F_{i}$ membership functions (see Figure 6) called MF-ij and each module MF-ij implements the *j*-th membership function associated with the *i*-th input, $\mu_{i,j}\left(x_{i}\left(n\right)\right)$. Each *n*-th time, all membership functions MF-ij are executed in parallel producing at the output a *N* bits unsigned signal of type uN.N, without the integer part, called $f_{i,j}\left[\mathrm{uN}.N\right]\left(n\right)$ (see Figure 6). The Fuzzy-PI controller proposed here uses $F_{0}+F_{1}$ membership functions.

**Figure 7:**
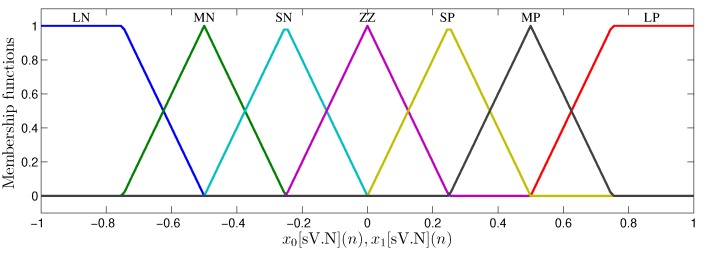
Membership functions from inputs $x_{0}\left[\mathrm{sV}.N\right]\left(n\right)$ and $x_{1}\left[\mathrm{sV}.N\right]\left(n\right)$.

Figure 7 shows the membership functions implemented in the MFM. For both variables, $x_{0}\left[\mathrm{sV}.N\right]\left(n\right)$ and $x_{1}\left[\mathrm{sV}.N\right]\left(n\right)$, seven functions of pertinence were created (trapezoidal at the ends and triangular at the middle). The terms associated with the membership functions are Large Negative (LN), Moderate Negative (MN), Small Negative (SN), Zero (ZZ), Small Positive (SP), Moderate Positive (MP) and Large Positive (LP).

Each *j*-th membership function associated with the *i*-th input was implemented directly on hardware based on the following expressions:

(12) $$\begin{array}{c} \mu_{i,j}^{RT}\left(x_{i}[\mathrm{sV}.N\right]\left(n\right))=\left\{\begin{array}{cc} 0 & \mathrm{if}\;x_{i}\left[\mathrm{sV}.N\right]\left(n\right)>d_{i,j}\left[\mathrm{sW}.T\right]\\ G_{i,j}^{RT}\left(n\right) & \mathrm{if}\;c_{i,j}\left[\mathrm{sW}.T\right]\leq x_{i}\left[\mathrm{sV}.N\right]\left(n\right)\leq d_{i,j}\left[\mathrm{sW}.T\right],\\ 1 & \mathrm{if}\;x_{i}\left[\mathrm{sV}.N\right]\left(n\right)<c_{i,j}\left[\mathrm{sW}.T\right] \end{array}\right. \end{array}$$

being $\mu_{i,j}^{RT}\left(\cdot \right)$ the trapezoidal function on the right, $c_{i,j}\left[\mathrm{sW}.T\right]$ and $d_{i,j}\left[\mathrm{sW}.T\right]$ are constants ($c_{i,j}\left[\mathrm{sW}.T\right]$ < $d_{i,j}\left[\mathrm{sW}.T\right]$) and

(13) $$G_{ij}^{RT}\left(n\right)=\frac{d_{i,j}\left[W.T\right]-x_{i}\left[\mathrm{sV}.N\right]\left(n\right)}{d_{i,j}\left[W.T\right]-c_{i,j}\left[W.T\right]},$$

where *W* is the total number of bits of the constants of the *j*-th activation function associated with *i*-th input and *T* is the number of bits of the fractional part.

The values of W and T will set the resolution of the activation functions. In the implementation proposed in this work, the value of W is always expressed as W=2×T+1.

For the left side trapezoidal $\mu_{i,j}^{LT}\left(\cdot \right)$, we have

(14) $$\begin{array}{c} \mu_{i,j}^{LT}\left(x_{i}[\mathrm{sV}.N\right]\left(n\right))=\left\{\begin{array}{cc} 0 & \;\mathrm{if}\;x_{i}\left[\mathrm{sV}.N\right]\left(n\right)<e_{i,j}\left[\mathrm{sW}.T\right]\\ G_{i,j}^{LT}\left(n\right) & \;\mathrm{if}\;e_{i,j}\left[\mathrm{sW}.T\right]\leq x_{i}\left[\mathrm{sV}.N\right]\left(n\right)\leq f_{i,j}\left[\mathrm{sW}.T\right],\\ 1 & \;\mathrm{if}\;x_{i}\left[\mathrm{sV}.N\right]\left(n\right)>f_{i,j}\left[\mathrm{sW}.T\right] \end{array}\right. \end{array}$$

with $\mu_{i,j}^{LT}\left(\cdot \right)$ the left trapezoidal function, $e_{i,j}\left[\mathrm{sW}.T\right]$ and $f_{i,j}\left[\mathrm{sW}.T\right]$ constants ($e_{i,j}\left[\mathrm{sW}.T\right]$ < $f_{i,j}\left[\mathrm{sW}.T\right]$) and

(15) $$G_{ij}^{LT}\left(n\right)=\frac{x_{i}\left[\mathrm{sV}.N\right]\left(n\right)-e_{i,j}\left[W.T\right]}{f_{i,j}\left[W.T\right]-e_{i,j}\left[W.T\right]}.$$

Triangular membership functions are expressed as

(16) $$\begin{array}{c} \mu_{i,j}^{T}\left(x_{i}[\mathrm{sV}.N\right]\left(n\right))=\left\{\begin{array}{cc} \mu_{i,j}^{LT}\left(x_{i}[\mathrm{sV}.N\right]\left(n\right)) & \mathrm{if}\;x_{i}\left[\mathrm{sV}.N\right]\left(n\right)<m_{i,j}\left[\mathrm{sW}.T\right]\\ \mu_{i,j}^{RT}\left(x_{i}[\mathrm{sV}.N\right]\left(n\right)) & \mathrm{if}\;x_{i}\left[\mathrm{sV}.N\right]\left(n\right)\geq m_{i,j}\left[\mathrm{sW}.T\right] \end{array}\right., \end{array}$$

where $m_{i,j}\left[\mathrm{sW}.T\right]$ is the triangle center point, that is, $m_{i,j}\left[\mathrm{sW}.T\right]=c_{i,j}\left[\mathrm{sW}.T\right]=f_{i,j}\left[\mathrm{sW}.T\right]$.

The values of W and T will set the resolution of the activation functions. In the implementation proposed in this work, the value of W is always expressed as W=2×T+1. Non-linear pertinence functions can be implemented with lookup tables (LUTs). Although this implementation uses only two inputs ($x_{0}\left[\mathrm{sV}.N\right]\left(n\right)$ and $x_{1}\left[\mathrm{sV}.N\right]\left(n\right)$) and seven membership functions for each input, this can be easily extended to more inputs and functions, since the entire implementation is performed in parallel.

#### 4.2.2. Operation Module (OM)

**Figure 8:**
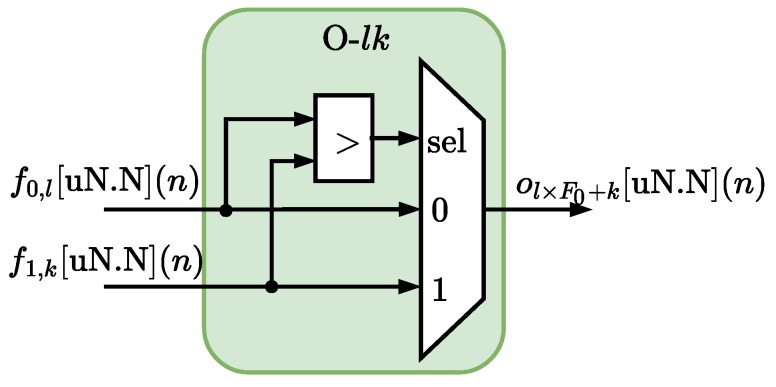
Arquitecture of the module O-lk associated with the operation between the fuzzyfied signal from the *l*-th membership function from input 0, $f_{0,l}\left[\mathrm{nN}.N\right]\left(n\right)$, with the *k*-th membership function from input 1, $f_{1,k}\left[\mathrm{uN}.N\right]\left(n\right)$ (see Equation (7)).

The $F_{0}+F_{1}$ outputs from the MFM module are passed to the OM module that performs all operations relative to the $F_{0}F_{1}$ rules, as described in Equation (6) in Section 3. Figure 8 details the hardware structure of one of the $F_{0}F_{1}$ operating modules, here called O-lk, which performs the minimum operation ("AND" connector) between the *l*-th membership function from input 0, $f_{0,l}\left[\mathrm{nN}.N\right]\left(n\right)$, with the *k*-th membership function from input 1, $f_{1,k}\left[\mathrm{uN}.N\right]\left(n\right)$ (see Equation (7)).

#### 4.2.3. Output Function Module (OFM)

**Figure 9:**
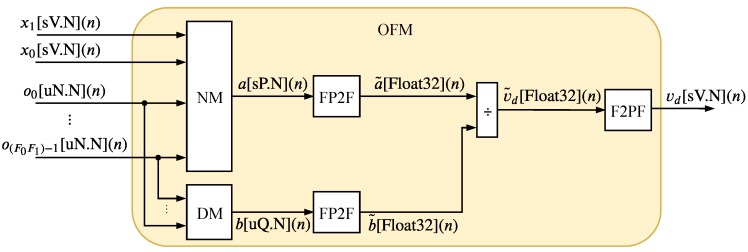
Hardware architecture of the OFM.

The OFM, illustrated in Figure 9, performs the generation of the TS-FIMM output variable during the step called defuzzification. This step essentially corresponds to the implementation of the Equation (7) presented in Section 3. The blocks called NM and DM perform the numerator and denominator operations presented in Equation (7), respectively.

**Figure 10:**
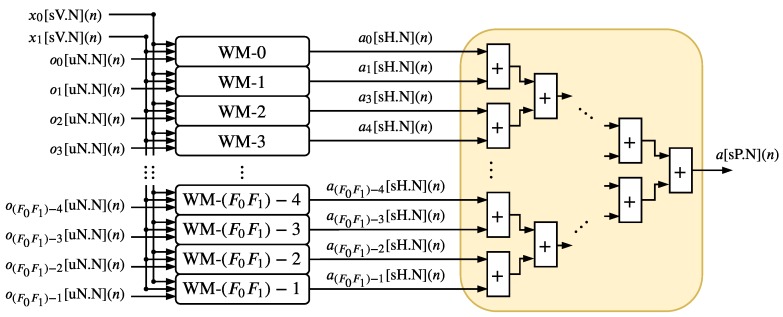
Hardware architecture of the NM.

**Figure 11:**
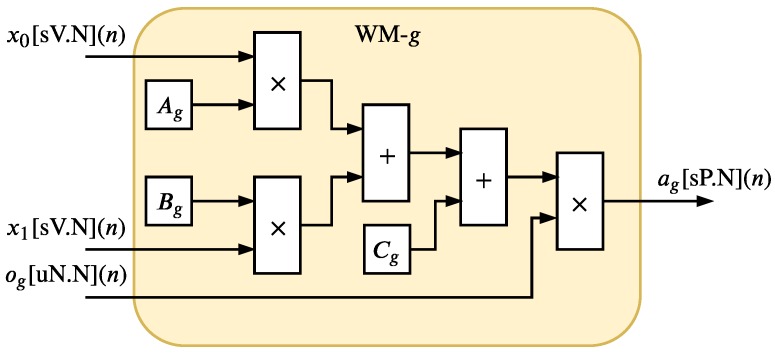
Hardware architecture of the WM-*g*.

Figure 10 and Figure 11 show the hardware implementation of the NM. The NM is composed of the $F_{0}F_{1}$ hardware components called WM-g and an adder tree structure. Each *g*-th WM-g, detailed in Figure 11, is a parallel hardware implementation of the variable $a_{g}$ presented in Equation (7). The $F_{0}F_{1}$ WMs hardware components are also implemented in parallel and they generated $F_{0}F_{1}$ signals $a_{g}\left[\mathrm{sH}.N\right]\left(n\right)$ in each *n*-th time instant. Since $-1<x_{0}\left[V.N\right]\left(n\right)<1$, $-1<x_{1}\left[V.N\right]\left(n\right)<1$, $0<o_{g}\left[\mathrm{uN}.N\right]\left(n\right)<1$, $-1<A_{g}<1$, $-1<B_{g}<1$ and $-1<C_{g}<1$ for $g=0,\ldots ,F_{0}F_{1}$, the variable *H* can be expressed as H=N+3.

The adder tree structure, illustrated in Figure 10, has a depth expressed as $\log_{2}\left(\left\lceil F_{0}F_{1}\right\rceil \right)$; thus, the output signal a(n) (see Equation (7)) can be performed as a[sP.N](n) where

(17) $$P=H+\log_{2}\left(\left\lceil F_{0}F_{1}\right\rceil \right).$$

**Figure 12:**
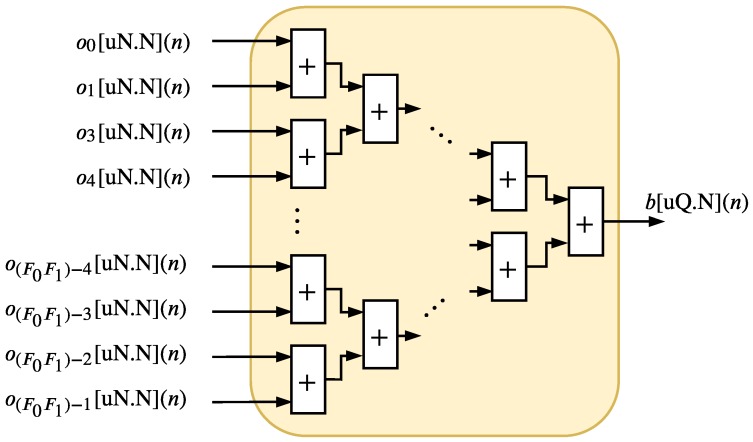
Hardware architecture of the DM.

The DM, presented in Figure 12, is characterized with an adder tree structure with depth also expressed as $\log_{2}\left(\left\lceil F_{0}F_{1}\right\rceil \right)$. The output signal of DM can be expressed as b[sQ.N](n) where

(18) $$Q=N+\log_{2}\left(\left\lceil F_{0}F_{1}\right\rceil \right)+1.$$

For the division calculation, the output signals, in fixed-point, of the NM and DM modules (a[sP.N](n) and b[sQ.N](n) are transformed to a 32-bit floating-point (IEEE754) standard by the Fixed-point to Float (FP2F) module ($\tilde{a}\left[\mathrm{Float}32\right]\left(n\right)$ e $\tilde{b}\left[\mathrm{Float}32\right]\left(n\right)$) and after division the TS-FIMM output is converted back into fixed-point by the Float to Fixed-point (F2FP) module.

Since the TS-FIMM inputs and the values of $A_{g}$, $B_{g}$ and $C_{g}$ are between −1 and 1, it can be guaranteed, from Equation (7), that the output, $v_{d}\left[\mathrm{sV}.N\right]\left(n\right)$, continue normalized between −1 and 1. Thus, one can use the same input resolution, that is, N for the fractional part and V=N+1 for the integer part, as shown in Figure 9.

### 4.3. Integration Module (IM)

**Figure 13:**
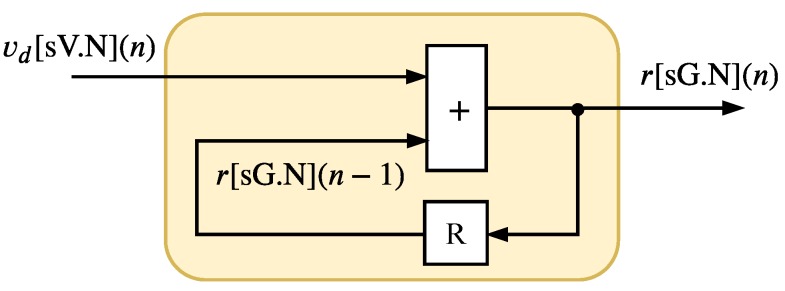
Hardware architecture of the IM.

The IM, shown in Figure 13, implements the Equation (9) presented in Section 3. This module is the last step on the Fuzzy-PI hardware, and it is composed of the accumulator with a saturation. The output signal, r(n), is expressed as r[sG.N](n), where

(19) $$G=N+\log_{2}\left(\left\lceil v_{\max}-v_{\min}\right\rceil \right)+1.$$

## 5. Synthesis Results

The synthesis results were obtained for a Fuzzy-PI controller (see Figure 2) and also to specific modules TS-FIMM-OS (see Figure 4) and TS-FIMM-P (see Figure 5). The separate synthesis of the TS-FIMM allows for analysis of the Fuzzy inference algorithm core in the complete hardware proposal. All synthesis results used an FPGA Xilinx Virtex 6 (6-bits LUTs) xc6vlx240t-1ff1156 and that has 301,440 registers, 150,720 logical cells to be used as LUTs and 768 multipliers.

### 5.1. Synthesis Results—TS-FIMM Hardware

Table 1 and Table 2 present the synthesis results related to hardware occupancy and the maximum throughput, $R_{s}=1/t_{s}$, in Mega samples per second (Msps) of the system for several values of *N* and *T*. Table 1 and Table 2 show the synthesis results associated with TS-FIMM-OS and TS-FIMM-P, respectively. The columns, NR, NLUT, and NMULT, represent the number of registers, logic cells used as LUTs, and multipliers in the hardware implemented in the FPGA, respectively. The PNR, PNLUT, and NMULT columns represent the percentage relative to the total FPGA resources.

Synthesis results show that the hardware proposal for TS-FIMM takes up a small hardware space of less than 1%, PR, in registers and less than 7% in LUTs, PLUT, of the FPGA (see Table 1 and Table 2). These results enable the use of several TS-FIMM implemented in parallel on FPGA, allowing for accelerating several applications in massive data environments. On the other hand, the low hardware consumption allows the use of TS-FIMM in small FPGAs of low cost and consumption for applications of IoT and M2M. Another important point to be analyzed, still in relation to the synthesis, is the linear behavior of the hardware consumption in relation to the number of bits, unlike the work presented in [45], and this is important, since it makes possible the use systems with higher resolution.

The values of throughput, $R_{s}$, were very relevant, with values about 11.5Msps for TS-FIMM-OS and values about 17Msps for TS-FIMM-P. These values enable its application in various large volume problems for processing as presented in [30] or in problems with fast control requirements such as tactile internet applications [21,22]. It is also observed that throughput has a linear behavior as a function of the number of bits.

The TS-FIMM-P with a speedup of about 1.47× ($\frac{17\mathrm{Msps}}{11.5\mathrm{Msps}}$) is related to the TS-FIMM-OS. This speedup was driven by the critical path reduction with the pipeline scheme. However, the pipeline scheme in TS-FIMM-P used about 3.4× registers (NR) more than TS-FIMM-OS.

**Figure 14:**
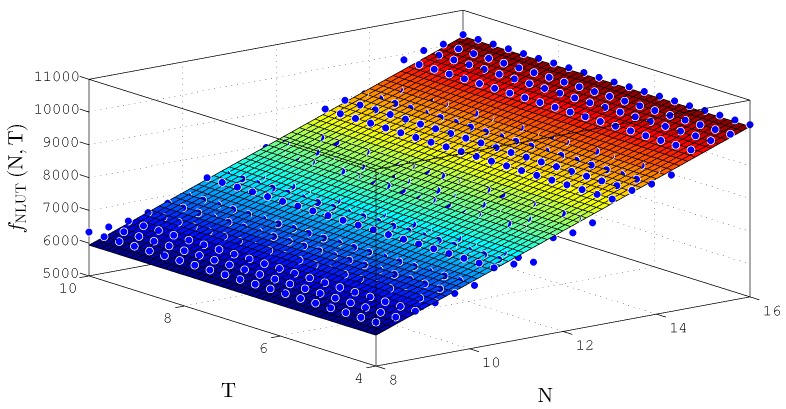
Plane, $f_{\mathrm{NLUT}}\left(N,T\right)$, found to estimate the number of LUTs as a function of the number of bits N and T for TS-FIMM-OS.

**Figure 15:**
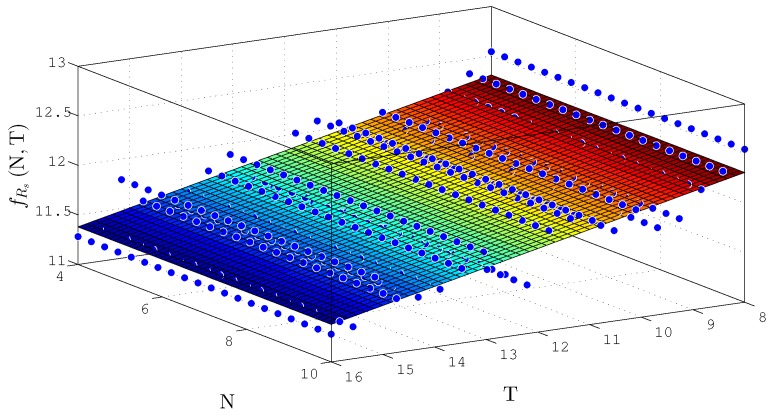
Plane, $f_{R_{s}}\left(N,T\right)$, found to estimate throughput, $R_{s}$, for different number of bits N and T for TS-FIMM-OS.

Figure 14 and Figure 15 show the behavior surfaces of the number of LUTs (NLUT) and throughput in function of N and T for TS-FIMM-OS, respectively. For both cases, an adjustment was made, through a regression technique, to find the plane that best matches the measured points. For the case of NLUT, the plane, $f_{\mathrm{NLUT}}\left(N,T\right)$, was expressed by

(20) $$f_{\mathrm{NLUT}}\left(N,T\right)\approx 1682+532.2\times N+6.493\times 10^{-13}\times T,$$

with $R^{2}=0.9766$. For throughput, in Msps, a plane was found, $f_{R_{s}}\left(N,T\right)$, characterized as

(21) $$f_{R_{s}}\left(N,T\right)\approx 13.24-0.1163\times N+3.414\times 10^{-16}\times T,$$

with $R^{2}=0.7521$.

**Figure 16:**
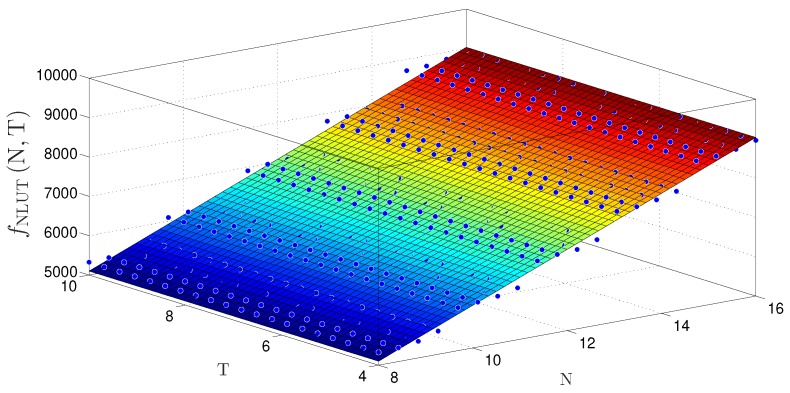
Plane, $f_{\mathrm{NLUT}}\left(N,T\right)$, found to estimate the number of LUTs as a function of the number of bits N and T for TS-FIMM-P.

**Figure 17:**
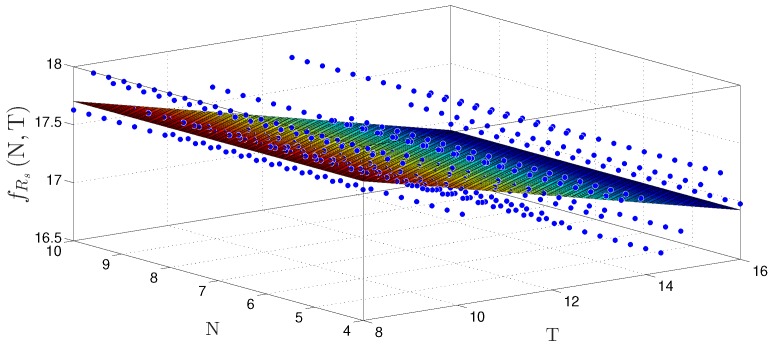
Plane, $f_{R_{s}}\left(N,T\right)$, found to estimate throughput, $R_{s}$, for different number of bits N and T for TS-FIMM-P.

The behavior surfaces of the number of LUTs (NLUT) and throughput in function of N and T for TS-FIMM-P are presented in Figure 16 and Figure 17, respectively. For the case of NLUT, the plane, $f_{\mathrm{NLUT}}\left(N,T\right)$, was expressed by

(22) $$f_{\mathrm{NLUT}}\left(N,T\right)\approx 1171+491.1\times N+4.245\times 10^{-13}\times T,$$

with a $R^{2}=0.9838$. For throughput in Msps, a plane was found, $f_{R_{s}}\left(N,T\right)$, characterized as

(23) $$f_{R_{s}}\left(N,T\right)\approx 18.48-0.09704\times N-5.365\times 10^{-16}\times T,$$

with $R^{2}=0.5366$.

### 5.2. Synthesis Results—Fuzzy-PI Controller Hardware

Table 3 and Table 4 present the synthesis results related to hardware occupancy and throughput, $R_{s}$, for the Fuzzy-PI controller hardware (see Figure 2). The results are presented for several values of *N* and T=10.

Synthesis results, drawn on Table 3 and Table 4, show that the proposed implementation requires a small fraction of hardware space, less than 1%, PR, in registers and less than 8% in LUTs, PLUT, of the FPGA. In addition, it is possible to see the numbers of embedded multipliers, PNMULT, remained below 7%. This occupation enables the use of several Fuzzy-PI controllers in parallel in the same FPGA hardware, and this allows various control systems running in parallel on industrial applications. The low size implementation also allows the use in low cost and power consumption IoT and M2M applications. Regarding throughput, $R_{s}$, the results obtained were highly relevant, with values between 15.33, and 13.41Msps, which enables its application in several problems with large data volume for processing as presented in [30] or in problems with fast control requirements such as tactile internet applications [21].

## 6. Validation Results

### 6.1. Validation Results—TS-FIMM Hardware

**Figure 18:**
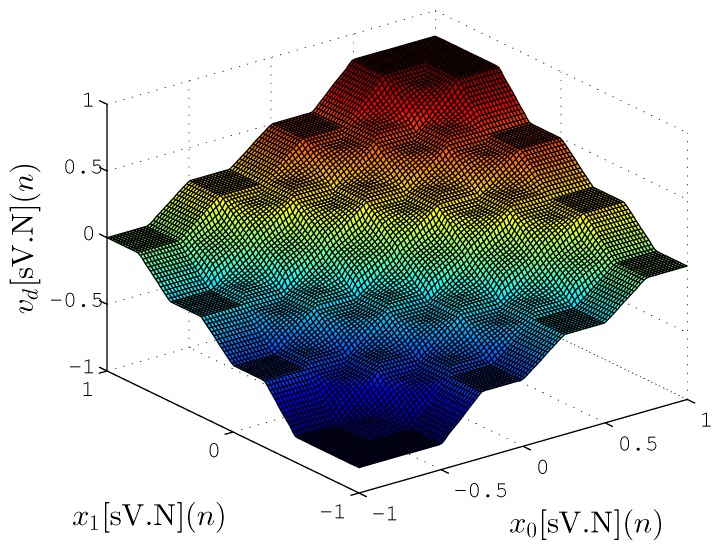
Mapping between input and output from TS-FIMM hardware using fixed-point with N=8, V=9 and T=4.

**Figure 19:**
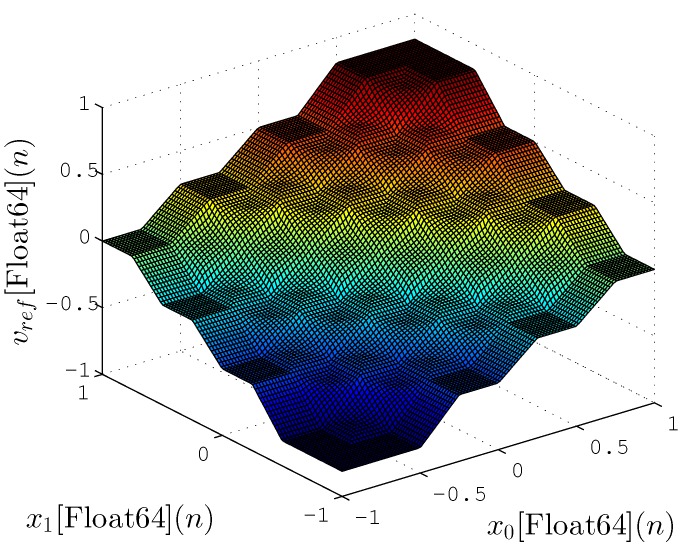
Mapping between input and ouput from TS-FIMM generated by Matlab Fuzzy Logic Toolbox using a double format.

Figure 18 and Figure 19 show the mapping between input ($x_{0}\left(n\right)$ and $x_{1}\left(n\right)$) and output $v_{d}\left(n\right)$ for proposed hardware and a reference implementation with Fuzzy Matlab Toolbox (License number 1080073) [46], respectively. The Matlab implementation, shown in Figure 19, uses floating-point format with 64 bits (double precision) while in Figure 18 the proposed hardware-generated mapping is presented using lower resolution synthesized (N=8, V=9 and T=4). These figures are able to present a qualitative representation of the proposed implementation, in which the obtained results are quite similar to those expected.

Table 5 shows the mean square error (MSE) between the Fuzzy Matlab Toolbox and the proposed hardware implementation for several cases *N* and *T*. For the experiment, the calculation of MSE is expressed as

(24) $$MSE=\frac{1}{Z}\sum_{n=0}^{Z-1}{\left(v_{ref}\left[\mathrm{Float}64\right]\left(n\right)-v_{d}\left[\mathrm{sV}.N\right]\left(n\right)\right)}^{2},$$

where *Z* represents the number of tested points that corresponded to 10,000 points spread evenly within the limits of the input values (−1 and 1). Figure 18 and Figure 19 were generated with these points.

The results obtained in relation to MSE were also quite significant, showing that the TS-FIMM hardware has a response quite similar to the implementation with 64 bits even for a fixed-point resolution of 8 bits ($MSE=2.395\times 10^{-6}$). Another interesting fact was related to the values of *T* that did not significantly influence the MSE value for the pertinence functions used (see Figure 7) in the project. It is important to note that the implementation of TS-FIMM hardware with few bits leads to smaller hardware, low-power consumption or high-throughput values.

### 6.2. Validation Results—Fuzzy-PI Controller Hardware

**Figure 20:**
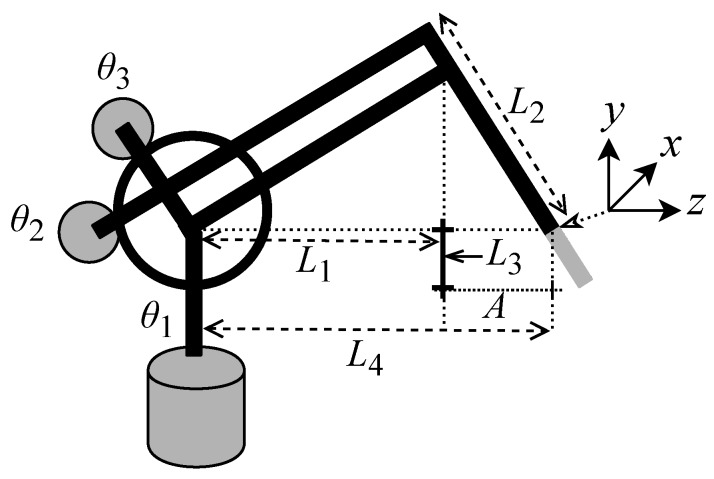
Structure of 3DOF Phantom Omni robotic manipulator.

In order to validate the results of the Fuzzy-PI controller in hardware, bit-precision simulation tests were performed with a nonlinear dynamic system characterized by a robotic manipulator system called the Phantom Omni [47,48,49,50]. The Phantom Omni is a 6-DOF (Degree Of Freedom) manipulator, with rotational joints. The first three joints are actuated, while the last three joints are non-actuated [50]. As illustrated in Figure 20, the device can be modeled as 3DOF robotic manipulator with two segments $L_{1}$ and $L_{2}$. The segments are interconnected by three rotary joints angles $\theta_{1}$, $\theta_{2}$, and $\theta_{3}$. The Phantom Omni has been widely used in literature, as presented in [47,48,49]. Simulations used $L_{1}=0.135\;\mathrm{mm}$, L2=L1, L3=0.025mm, and L4=L1+A, where A=0.035mm as described in [49].

Nonlinear, second order, ordinary differential equation used to describe the dynamics of the Phantom Omni can be expressed as

(25) $$M\left(\theta (t)\right)\ddot{\theta }\left(t\right)+C\left(\theta \left(t\right),\dot{\theta }\left(t\right)\right)\dot{\theta }\left(t\right)+g\left(\theta (t)\right)-f\left(\dot{\theta }\left(t\right)\right)=\tau \left(t\right)$$

where θ(t) is the vector of joints expressed as

(26) $$\theta \left(t\right)={\left[\begin{array}{ccc} \theta_{1}\left(t\right) & \theta_{2}\left(t\right) & \theta_{3}\left(t\right) \end{array}\right]}^{T}\in R^{3\times 1},$$

τ is the vector of torques acting expressed as

(27) $$\tau \left(t\right)={\left[\begin{array}{ccc} \tau_{1}\left(t\right) & \tau_{2}\left(t\right) & \tau_{3}\left(t\right) \end{array}\right]}^{T}\in R^{3\times 1}.$$

$M\left(\theta (t)\right)\in R^{3\times 3}$ is the inertia matrix, $C\left(\theta \left(t\right),\dot{\theta }\left(t\right)\right)\in R^{3\times 3}$ is the Coriolis and centrifugal forces matrix, $g\left(\theta (t)\right)\in R^{3\times 1}$ represents the gravity force acting on the joints, θ(t), and the $f\left(\dot{\theta }\left(t\right)\right)$ is the friction force on the joints, θ(t) [47,48,49,50].

**Figure 21:**
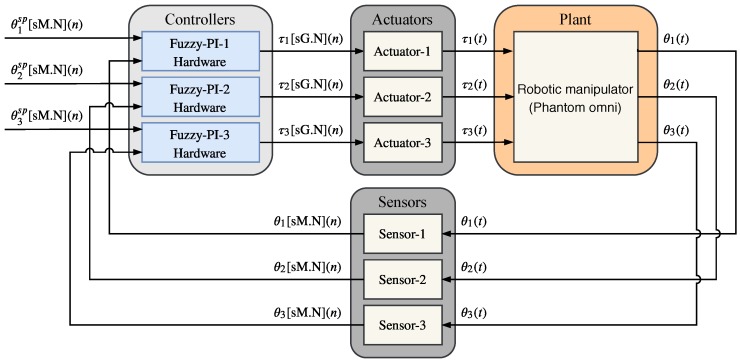
Simulated system used to validate the Fuzzy-PI hardware proposal. The plant is the 3DOF Phantom Omni robotic manipulator and there are three pieces of Fuzzy-PI hardware running in parallel.

Figure 21 shows the simulated system where the plant is the 3DOF Phantom Omni robotic manipulator. The controlled variables are the angular position of the joints $\theta_{1}$, $\theta_{2}$, and $\theta_{3}$ and the actuator variables are the torques $\tau_{1}$, $\tau_{2}$, and $\tau_{3}$. The control system has three angular position sensors and each *i*-th Sensor−i convert the *i*-th continuous angle signal, $\theta_{i}\left(t\right)$ to discrete angle signal, $\theta_{i}\left(n\right)$. There are three pieces of Fuzzy-PI hardware running in parallel and every *i*-th Sensor−i is connected with Fuzzy-PI hardware, Fuzzy−PI−i. Each piece of *i*-th Fuzzy−PI−i hardware generates the *i*-th discrete torques acting signal, $\tau_{i}\left(n\right)$, and every *i*-th discrete torque signal, $\tau_{i}\left(n\right)$, is connected to *i*-th actuator, Actuator−i. Finally, each *i*-th actuator, Actuator−i, generates the *i*-th continuous torque signal, $\tau_{i}\left(t\right)$ to the applied on the robotic manipulator. The set point variables (or reference signal) are an angular position of the joints, and they are expressed by $\theta_{1}^{sp}\left(n\right)$, $\theta_{2}^{sp}\left(n\right)$ and $\theta_{3}^{sp}\left(n\right)$.

**Figure 22:**
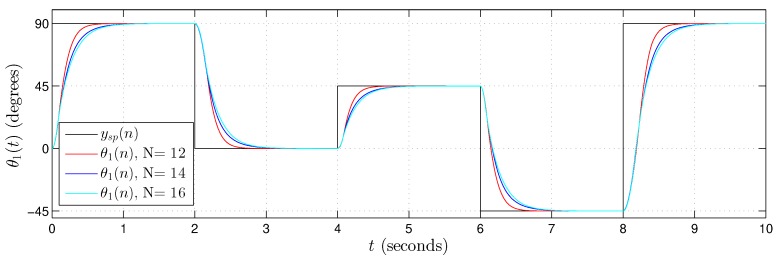
Validation results from the proposed Takagi–Sugeno Fuzzy-PI hardware. Simulation trajectory for $\theta_{1}\left(t\right)$ with $\theta_{1}\left(n\right)$ using N={12,14,16} bits in the fractional part.

**Figure 23:**
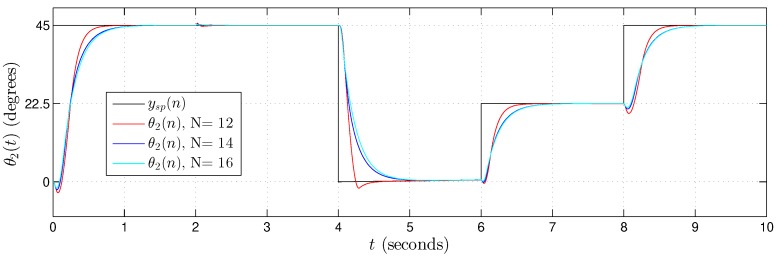
Validation results from the proposed Takagi–Sugeno Fuzzy-PI hardware. Simulation trajectory for $\theta_{2}\left(t\right)$ with $\theta_{2}\left(n\right)$ using N={12,14,16} bits in the fractional part.

**Figure 24:**
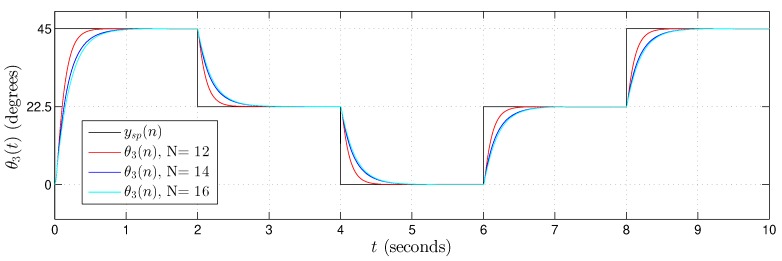
Validation results from the proposed Takagi–Sugeno Fuzzy-PI hardware. Simulation trajectory for $\theta_{3}\left(t\right)$ with $\theta_{3}\left(n\right)$ using N={12,14,16} bits in the fractional part.

Figure 22, Figure 23 and Figure 24 present the hardware validation results for various resolutions in terms of the number of bits of the fractional part, N={12,14,16} for discrete controlled variables $\theta_{1}\left(n\right)$, $\theta_{2}\left(n\right)$ and $\theta_{3}\left(n\right)$, respectively. The simulation trajectory was of 10 seconds and every 2 seconds was changing. Table 6 shows the angle trajectory changing for set point variables $\theta_{1}^{sp}\left(n\right)$, $\theta_{2}^{sp}\left(n\right)$ and $\theta_{3}^{sp}\left(n\right)$. Simulations used $t_{s}=1\times 10^{-5}$, Kp=2000 and Ki=0.1 for each *i*-th Fuzzy−PI−i hardware.

In the results presented in Figure 22, Figure 23 and Figure 24, it is possible to observe that the controller followed the plant reference in all cases. Results also showed that the Takagi–Sugeno Fuzzy-PI hardware proposal has been following the reference even for a small amount of bits, that is, a low resolution.

## 7. Comparison with Other Works

### 7.1. Throughput Comparison

Table 7 shows a comparison with other works in the literature. Parameters like inference machine (IM) type (Takagi–Sugeno or Mamdani), number of inputs (NI), number of rules (NR), number of outputs (NO), number of bits (NB), throughput in Msps, $R_{s}$ and Mflips (Mega fuzzy logic inference per second) are showed. In additional, Table 7 also shows the speedups (in Msps and Mflips) achieved of the TS-FIMM-OS, TS-FIMM-P, Fuzzy-PI controller with TS-FIMM-OS (Fuzzy-PI-OS) and with TS-FIMM-P (Fuzzy-PI-P) over the other works in the literature. The value in flips can be calculated as $\mathrm{NR}\times R_{s}$.

In the work presented in [11], the results were obtained for several cases and, for one with two inputs, 35 rules, and one output (vehicle parking problem), the proposed hardware achieved a maximum clock about 66.251MHz with 10 bits [12,13]. However, the FIM takes 10 clocks to complete the inference step; in other words, the hardware proposal in [11] achieves a throughput in Msps of about $\frac{66.251}{10}\approx 6.63\;\mathrm{Msps}$ and in Mflips of about 6.63×35≈232.05Mflips. The speedup in Msps for the TS-FIMM-OS, TS-FIMM-P, Fuzzy-PI-OS, and Fuzzy-PI-P are $\frac{12.05\;\mathrm{Msps}}{6.63\;\mathrm{Msps}}\approx 1.82$, $\frac{17.63\;\mathrm{Msps}}{6.63\;\mathrm{Msps}}\approx 2.66$, $\frac{10.16\;\mathrm{Msps}}{6.63\;\mathrm{Msps}}\approx 1.53$, and $\frac{13.86\;\mathrm{Msps}}{6.63\;\mathrm{Msps}}\approx 2.09$, respectively. As the hardware proposal in this paper used 49 rules, the speedup in Mflips can be calculated as the throughput in Msps $\times \frac{49}{35}$ that is, the speedup for the TS-FIMM-OS, TS-FIMM-P, Fuzzy-PI-OS and Fuzzy-PI-P are 1.82×1.4≈2.55, 1.82×1.4≈3.72, 1.53×1.4≈2.14, and 2.09×1.4≈2.93, respectively.

The work presented in [5] proposes a Takagi–Sugeno fuzzy controller on FPGA with two inputs, six rules, and three outputs. The hardware achieved a throughput of about 1Msps with 8 bits on the bus. With 8 bits, the speedup in Msps for the TS-FIMM-OS, TS-FIMM-P, Fuzzy-PI-OS, and Fuzzy-PI-P are $\frac{11.94\;\mathrm{Msps}}{1\;\mathrm{Msps}}\approx 11.94$, $\frac{17.55\;\mathrm{Msps}}{1\;\mathrm{Msps}}\approx 17.55$, $\frac{10.77\;\mathrm{Msps}}{1\;\mathrm{Msps}}\approx 10.77$, and $\frac{15.13\;\mathrm{Msps}}{1\;\mathrm{Msps}}\approx 15.13$, respectively. The speedup in Mflips is about $\frac{49}{6}\approx 8.16\times$ over the speedup in Msps.

In [16], a Mamdani fuzzy logic controller on FPGA was proposed. The hardware carries out a throughput of about 25Mflips with two inputs, 49 rules, one output, and 16 bits. Using 16 bits, the speedup in Mflips for the TS-FIMM-OS, TS-FIMM-P, Fuzzy-PI-OS, and Fuzzy-PI-P are $\frac{11.28\times 49\;\mathrm{Mflips}}{25\;\mathrm{Mflips}}\approx 22.11$, $\frac{16.98\times 49\;\mathrm{Mflips}}{25\;\mathrm{Mflips}}\approx 33.28$, $\frac{9.59\times 49\;\mathrm{Mflips}}{25\;\mathrm{Mflips}}\approx 18.79$, and $\frac{13.41\times 49\;\mathrm{Mflips}}{25\;\mathrm{Mflips}}\approx 26.28$, respectively. As the number of rules is 49, the speedup in Msps is equal to Mflips.

The work presented in [31] uses a Mamdani inference machine and the throughput in Mflips is about 48.23Mflips. The hardware designed in [31] operated with 8 bits, four inputs, nine rules, and one output. The speedup in Mflips, with 8 bits, for the TS-FIMM-OS, TS-FIMM-P, Fuzzy-PI-OS, and Fuzzy-PI-P are $\frac{11.94\times 49\;\mathrm{Mflips}}{48.23\;\mathrm{Mflips}}\approx 12.13$, $\frac{17.55\times 49\;\mathrm{Mflips}}{48.23\;\mathrm{Mflips}}\approx 17.83$, $\frac{10.77\times 49\;\mathrm{Mflips}}{48.23\;\mathrm{Mflips}}\approx 10.94$, and $\frac{13.41\times 49\;\mathrm{Mflips}}{48.23\;\mathrm{Mflips}}\approx 15.37$, respectively. The speedup in Msps is about $\frac{9}{49}\approx 0.18\times$ over the speedup in Mflips.

The hardware used in [14] takes six clock cycles over 10MHz (in four states) to execute a M-IM with 16 bits. This is equivalent to a throughput of about $\frac{10\;\mathrm{MHz}}{6}\approx 1.67\;\mathrm{Msps}$. The scheme proposed in [14] used two inputs, 25 rules, and one output. The speedup in Msps for the TS-FIMM-OS, TS-FIMM-P, Fuzzy-PI-OS, and Fuzzy-PI-P are $\frac{11.28\;\mathrm{Msps}}{1.67\;\mathrm{Msps}}\approx 6.75$, $\frac{16.98\;\mathrm{Msps}}{1.67\;\mathrm{Msps}}\approx 10.17$, $\frac{9.59\;\mathrm{Msps}}{1.67\;\mathrm{Msps}}\approx 5.74$, and $\frac{13.41\;\mathrm{Msps}}{1.67\;\mathrm{Msps}}\approx 8.03$, respectively. The speedup in Mflips is about $\frac{49}{25}\approx 1.96\times$ over the speedup in Msps.

The works presented in [18,20] show that a piece of hardware can achieve about 1Msps. The work presented in [18] uses two inputs, 25 rules, one output, and 8 bits, and the designer presented in [20] was projected with three inputs, 42 rules, and one output. The speedup in Msps for the TS-FIMM-OS, TS-FIMM-P, Fuzzy-PI-OS, and Fuzzy-PI-P are equal to previously calculated values used in [5]. The speedups in Mflips are about $\frac{49}{25}\approx 1.96\times$ and $\frac{49}{42}\approx 1.16\times$ over the speedup in Msps for works [18] and [20], respectively.

The hardware proposes in [7] achieved a throughput of about 1.56Msps with three inputs, two outputs, and 24 bits. The speedup in Msps for the TS-FIMM-OS, TS-FIMM-P, Fuzzy-PI-OS, and Fuzzy-PI-P are $\frac{11.28\;\mathrm{Msps}}{1.56\;\mathrm{Msps}}\approx 7.23$, $\frac{16.98\;\mathrm{Msps}}{1.56\;\mathrm{Msps}}\approx 10.88$, $\frac{9.59\;\mathrm{Msps}}{1.56\;\mathrm{Msps}}\approx 6.15$, and $\frac{13.41\;\mathrm{Msps}}{1.56\;\mathrm{Msps}}\approx 8.59$, respectively. The fuzzy system proposed in [7] does not use linguistic fuzzy rules, and it cannot calculate the throughput in Mflips.

There are multiple differences between the devices used for comparison, starting with the number of bits in the LUTs (4-bits LUTs [5,11], 5-bits LUTs [16], and 6-bit LUTs [7,14,18,20,31]), board manufacturer (Altera [5,16] and Xilinx [7,14,18,20,31]), and families used (Spartan-3A [5], Cyclone-II [11], Arria-V GX [16], Spartan-6 [18,20], Virtex-5 [7], and Virtex-7 [7] ). However, these differences have no significant influence on the throughput; the transmission rates of storage elements, such as LUTs, are in most cases of the same order of magnitude for devices using the same or similar technology. FPGAs have dedicated wires (called carry chains) between neighboring LUTs, and these circuits have a fast transmission rate that allows combining multiple LUTs [51,52]. Therefore, differences in size of LUTs do not significantly affect throughput. Unlike the referenced works, for which most use a serial structure, in this work, we use a completely parallel approach. Thus, the design of the hardware architecture is primarily responsible for the resulting performance.

### 7.2. Hardware Occupation Comparison

Table 8 shows a comparison regarding the hardware occupation between the proposed hardware in this work and other literature works presented in Table 7. The second, third, fourth, and fifth columns show the type of FPGA, the number of logic cells (NLC), the number of multipliers (NMULT), and the number of bits in memory block RAMs (NBitsM), respectively, and the last three columns show the ratio of the hardware occupation between the proposal presented here, $N_{\mathrm{hardware}}^{\mathrm{work}}$, and literature works, $N_{\mathrm{hardware}}^{\mathrm{ref}}$, presented in Table 7. The ratio of the hardware occupation can be expressed as

(28) $$R_{\mathrm{occupation}}=\left\{\begin{array}{cc} \frac{N_{\mathrm{hardware}}^{\mathrm{work}}}{N_{\mathrm{hardware}}^{\mathrm{ref}}} & ,\;\mathrm{for}\;N_{\mathrm{hardware}}^{\mathrm{work}}>0\;\mathrm{and}\;N_{\mathrm{hardware}}^{\mathrm{ref}}>0\\ \;\\ \frac{1}{N_{\mathrm{hardware}}^{\mathrm{ref}}} & ,\;\mathrm{for}\;N_{\mathrm{hardware}}^{\mathrm{work}}=0\;\mathrm{and}\;N_{\mathrm{hardware}}^{\mathrm{ref}}>0\\ \;\\ N_{\mathrm{hardware}}^{\mathrm{work}} & ,\;\mathrm{for}\;N_{\mathrm{hardware}}^{\mathrm{work}}>0\;\mathrm{and}\;N_{\mathrm{hardware}}^{\mathrm{ref}}=0\\ \;\\ 1 & ,\;\mathrm{for}\;N_{\mathrm{hardware}}^{\mathrm{work}}=0\;\mathrm{and}\;N_{\mathrm{hardware}}^{\mathrm{ref}}=0 \end{array}\right.,$$

where $N_{\mathrm{hardware}}^{\mathrm{work}}$ and $N_{\mathrm{hardware}}^{\mathrm{ref}}$ can be replaced by NLC, NMULT, or NBitsM.

The work presented in [11] used a Spartan 3A DSP FPGA from Xilinx, and it has a hardware occupation of about 199 slices, four multipliers, and one block RAM. As this FPGA uses about 2.25 LC per slice, it used about 447 LC and it has 1512K bits per block RAM. The scheme proposed in [5] used a Cyclone II EP2C35F672C6 FPGA from Intel, and it has a hardware occupation of about 1622 logic cells and 8.19 Kbits of memory. The EP2C35 FPGA has 105 block RAM and 4096 memory bits per block (4608 bits per block including 512 parity bits).

In [16], the work assigns an Arria V GX 5AGXFB3H4F40C5NES FPGA from Intel and it has a hardware occupation of about 3248 ALMs and 6.592 Kbits of memory. The Arria V GX 5AGX has two combinational logic cells per ALM. The hardware proposed in [31] employs a Spartan 6 FPGA from Xilinx, and it has a hardware occupation of about 544 LUTs and 32 multipliers. As this FPGA uses about 1.6 LC per LUT, it used about 447 LC.

The hardware presented in the manuscript [14] utilizes a Spartan 6 FPGA from Xilinx, and it has a hardware occupation of about 1802 slices and five multipliers. As this FPGA works with 6.34 LC per slice, it used about 11,425 LC. The proposal described in [20] take advantage of Virtex 5 xc5vfx70t-3ff1136 FPGA from Xilinx, and it has a hardware occupation of about 8195 LUTs and 53 multipliers. As this FPGA uses about 1.6 LC per LUT, it used about 13,108 LC. For 6-input LUT, they use the multiplier 1.6. The work presented in [7] used a Virtex 7 VX485T-2 FPGA from Xilinx, and it has a hardware occupation of about 1948 slices and 38 multipliers. As this FPGA uses about 6.4 LC per slice, it used about 12,468 LC.

Regarding hardware utilization, the size in bits of the LUTs can have influence when comparing the NLCs in different FPGAs. Since the Virtex 6 (the FPGA board used in this work) has 6-bit LUTs, we can apply a relation factor 4/6 to compare between 4-bit and 6-bit LUTs and 5/6 for comparisons between 5-bit and 6-bit LUTs. In the case of 4-bit LUTs (the works presented in [5,11]), the NLC is reduced by 4/6 and the hardware utilization ratio, $R_{\mathrm{occupation}}$, increases by 34%. For 5-bit LUTs (the work presented in [16]), the NLC is reduced by 5/6 and the $R_{\mathrm{occupation}}$ increases by 17%.

### 7.3. Power Consumption Comparison

Table 9 shows the dynamic power saving regarding the dynamic power. The dynamic power can be expressed as

(29) $$P_{d}\propto N_{g}\times F_{\mathrm{clk}}\times V_{DD}^{2},$$

where $N_{g}$ is the number of elements (or gates), $F_{\mathrm{clk}}$ is the maximum clock frequency, and $V_{DD}$ is the supply voltage. The frequency dependence is more severe than Equation (31) suggests, given that the frequency at which a CMOS circuit can operate is approximately proportional to the voltage [41]. Thus, the dynamic power can be expressed as

(30) $$P_{d}\propto N_{g}\times F_{\mathrm{clk}}^{3}.$$

For all comparisons, the number of elements, $N_{g}$, was calculated as

(31) $$N_{g}=\mathrm{NLC}+\mathrm{NMULT}.$$

Based on Equation (30), the dynamic power saving can be expressed as

(32) $$S_{d}=\frac{N_{g}^{\mathrm{ref}}\times {\left(F_{\mathrm{clk}}^{\mathrm{ref}}\right)}^{3}}{N_{g}^{\mathrm{work}}\times {\left(F_{\mathrm{clk}}^{\mathrm{work}}\right)}^{3}},$$

where the $N_{g}^{\mathrm{ref}}$ and $F_{\mathrm{clk}}^{\mathrm{ref}}$ are the number of elements (NLC+NMULT) and the maximum clock frequency of the literature works, respectively, and the $N_{g}^{\mathrm{work}}$ and $F_{\mathrm{clk}}^{\mathrm{work}}$ are the number of elements (NLC+NMULT) and the maximum clock frequency of this work, respectively. Differently from the literature, the hardware proposed here uses a fully parallelization layout, and it spends a one clock cycle per sample processing. In other words, the maximum clock frequency is equivalent to the throughput, $F_{\mathrm{clk}}^{\mathrm{work}}\equiv R_{s}$.

With the exception of the Spartan-3A (presented in [11]), which uses 4-bit LUTs and the Arria-V GX (presented in [16]), which uses 5-bit LUTs, the other devices used for power analysis have 6-bit LUTs such as the Virtex-6. Thus, as indicated previously (see Section 7.2), in the case of the Spartan-3A and the Arria-V GX, the NLC value is recalculated using a 6-bit LUT as reference. For the Spartan-3A, the NLC becomes $451\times \frac{4}{6}\approx 301$, with a dynamic power saving of approximately ≈25×. For the Arria-V GX, the NLC becomes $6496\times \frac{5}{6}\approx 5413$, with a dynamic power saving of approximately $\approx 10^{6}\times$. However, according to Equation (30), this reduction of NLCs will not have a significant impact on the dynamic power saving since it increases with frequency cube.

### 7.4. Analysis of the Comparisons

Results presented in Table 7 and Table 9 demonstrate that the fully parallelization strategy adopted here can achieve significant speedups and power consumption reductions. On the other hand, the fully parallelization scheme can increase the hardware consumption, see Table 8.

The mean value of speedup was about 10.89× in Msps and 30.89× in Mflips (see Table 7) and this results are very expressive to big data and MMD applications [1,2,3]. High-throughput fuzzy controllers are also important for speed control systems such as tactile internet applications [21,22].

This manuscript proposal has LC resources with higher utilization than the literature proposals (Table 8). The mean value regarding NLC utilization was about 6.89×; in other words, the fuzzy hardware scheme proposed here has used 6.89× more LC than the literature proposals. In the case of multipliers (NMULT), the mean value of the additional hardware was about 17.69×. Despite being large relative values, Table 1, Table 2, Table 3 and Table 4 show that the fuzzy hardware proposals in this work expend no more than 7% of the FPGA resource. Another important aspect is the block RAM resource utilization (NBitsM). The fully parallel computing scheme proposed here does not spend clock time to access information in block RAM, and this can increase the throughput and decrease the power consumption (see [5,11,16] in Table 7, Table 8 and Table 9).

The fully parallel designer allows for executing many operations per clock period, and this reduces the clock frequency operation and increases the throughput. Due to the nonlinear relationship with clock frequency operation (see Equation (30)), this strategy permits a considerable reduction of the dynamic power consumption (see Table 9). The results presented in Table 9 show that the power saving can achieve values from 4 until $10^{6}$ times, and these results are quite significant and enable the use of the proposed hardware here in several IoT applications.

## 8. Conclusions

This work aimed to develop a hardware dedicated to the fuzzy inference machine, the Takagi–Sugeno Fuzzy-PI controller. The developed hardware used a fully parallel implementation with fixed- and floating-point representations in distinct parts of the proposed scheme. All the details of the implementation were presented as well as the synthesis results and the bit-precision simulations. The synthesis was performed for several bit size resolutions and showed that the proposed hardware is viable for use in applications with critical processing time requirements. In order to characterize the proposed hardware, curves were generated, using the synthesis data obtained, to predict hardware consumption and throughput for all bit sizes. In addition, comparison results concerning throughput, hardware occupation, and power saving with other literature proposals were presented. 

## Figures and Tables

**Table 1:** Synthesis results (hardware requirement and time) associated with TS-FIMM-OS hardware.

N	T	NR	PR	NLUT	PLUT	NMULT	PNMULT	$t_{s}$ (ns)	$R_{s}$ (Msps)
8	4	217	≈0.07%	6339	≈4.21%	49	≈6.38%	79.72	12.54
	6			6381	≈4.23%			80.95	12.35
	8			6452	≈4.28%			81.96	12.20
	10			6598	≈4.38%			83.76	11.94
10	4	259	≈0.09%	6772	≈4.49%	49	≈6.38%	84.18	11.88
	6			6904	≈4.58%			82.70	12.09
	8			7331	≈4.86%			83.94	11.91
	10			7331	≈4.86%			83.00	12.05
12	4	324	≈0.11%	7280	≈4.83%	49	≈6.38%	82.65	12.10
	6			7916	≈5.25%			83.28	12.01
	8			7954	≈5.28%			87.02	11.49
	10			8147	≈5.41%			85.99	11.63
14	4	384	≈0.13%	8761	≈5.81%	49	≈6.38%	84.12	11.89
	6			8915	≈5.91%			85.08	11.75
	8			8999	≈5.97%			86.39	11.58
	10			9163	≈6.08%			86.75	11.53
16	4	428	≈0.14%	9816	≈6.51%	49	≈6.38%	86.42	11.54
	6			9990	≈6.63%			84.80	11.79
	8			10,072	≈6.68%			88.31	11.32
	10			10,252	≈6.80%			88.65	11.28

**Table 2:** Synthesis results (hardware requirement and time) associated with TS-FIMM-P hardware.

N	T	NR	PR	NLUT	PLUT	NMULT	PNMULT	$t_{s}$ (ns)	$R_{s}$ (Msps)
8	4	746	≈0.25%	5326	≈3.53%	49	≈6.38%	56.73	17.62
	6			5350	≈3.55%			55.81	17.92
	8			5422	≈3.60%			56.18	17.80
	10			5590	≈3.71%			56.97	17.55
10	4	917	≈0.30%	6093	≈4.04%	49	≈6.38%	57.21	17.48
	6			6141	≈4.07%			57.88	17.28
	8			6199	≈4.11%			57.63	17.35
	10			6317	≈4.19%			56.72	17.63
12	4	1113	≈0.37%	6910	≈4.58%	49	≈6.38%	57.90	17.27
	6			6982	≈4.63%			58.22	17.18
	8			7016	≈4.65%			58.60	17.06
	10			7172	≈4.76%			56.26	17.77
14	4	1301	≈0.43%	7799	≈5.17%	49	≈6.38%	58.60	17.06
	6			7823	≈5.19%			58.22	17.18
	8			7905	≈5.24%			58.26	17.16
	10			8031	≈5.33%			60.00	16.66
16	4	1477	≈0.49%	8713	≈5.78%	49	≈6.38%	59.43	16.83
	6			8737	≈5.80%			58.14	17.20
	8			8819	≈5.85%			57.89	17.27
	10			8955	≈5.94%			58.90	16.98

**Table 3:** Synthesis results (hardware requirement and time) associated with Fuzzy-PI controller hardware with TS-FIMM-OS.

N	NR	PR	NLUT	PLUT	NMULT	PNMULT	$t_{s}$ (ns)	$R_{s}$ (Msps)
8	261	≈0.09%	6834	≈4.53%	49	≈6.38%	92.87	10.77
10	307	≈0.10%	7331	≈4.86%	49	≈6.38%	98.44	10.16
12	375	≈0.12%	8409	≈5.58%	49	≈6.38%	98.68	10.13
14	438	≈0.15%	9460	≈6.28%	49	≈6.38%	99.98	10.00
16	488	≈0.16%	10,595	≈7.03%	49	≈6.38%	104.31	9.59

**Table 4:** Synthesis results (hardware requirement and time) associated with Fuzzy-PI controller hardware with TS-FIMM-P.

N	NR	PR	NLUT	PLUT	NMULT	PNMULT	$t_{s}$ (ns)	$R_{s}$ (Msps)
8	790	≈0.26%	5826	≈3.87%	49	≈6.38%	66.08	15.13
10	965	≈0.32%	6317	≈4.19%	49	≈6.38%	72.16	13.86
12	1164	≈0.39%	7434	≈4.93%	49	≈6.38%	68.95	14.50
14	1355	≈0.45%	8328	≈5.53%	49	≈6.38%	73.23	13.66
16	1537	≈0.51%	9298	≈6.17%	49	≈6.38%	74.56	13.41

**Table 5:** Mean square error (MSE) between the Fuzzy Matlab Toolbox and the proposed hardware implementation for several cases *N* and *T*.

N	T	MSE (See Equation (24))
8	4	$2.4\times 10^{-6}$
	6	
	8	
	10	
10	4	$1.3\times 10^{-7}$
	6	
	8	
	10	
12	4	$7.2\times 10^{-9}$
	6	
	8	
	10	
14	4	$4.9\times 10^{-10}$
	6	
	8	
	10	
16	4	$2.7\times 10^{-11}$
	6	
	8	
	10	

**Table 6:** Angle trajectory changing for set point variables $\theta_{1}^{sp}\left(n\right)$, $\theta_{2}^{sp}\left(n\right)$ and $\theta_{3}^{sp}\left(n\right)$.

Set Point	0−2s	2s−4s	4s−6s	6s−8s	8s−10s
$\theta_{1}^{sp}\left(n\right)$ (Figure 22)	90°	0°	45°	−45°	90°
$\theta_{2}^{sp}\left(n\right)$ (Figure 23)	45°	45°	0°	22.5°	45°
$\theta_{3}^{sp}\left(n\right)$ (Figure 24)	45°	22.5°	0°	22.5°	45°

**Table 7:** Throughput comparison with other works.

References	IM	NI	NR	NO	NB	Msps	Mflips	This Work	Speedup	
									Msps	Mflips
[11] (2013)	TS-IM	2	35	1	10	≈6.63	≈232.05	TS-FIMM-OS	≈1.82×	≈2.55×
								TS-FIMM-P	≈2.66×	≈3.72×
								Fuzzy-PI-OS	≈1.53×	≈2.14×
								Fuzzy-PI-P	≈2.09×	≈2.93×
[5] (2014)	TS-IM	2	6	3	8	≈1.00	≈6.00	TS-FIMM-OS	≈11.94×	≈97.43×
								TS-FIMM-P	≈17.55×	≈143.20×
								Fuzzy-PI-OS	≈10.77×	≈87.88×
								Fuzzy-PI-P	≈15.13×	≈123.46×
[16] (2015)	M-IM	2	49	1	16	≈0.51	≈25.00	TS-FIMM-OS	≈22.11×	≈22.11×
								TS-FIMM-P	≈33.28×	≈33.28×
								Fuzzy-PI-OS	≈18.79×	≈18.79×
								Fuzzy-PI-P	≈26.28×	≈26.28×
[31] (2016)	M-IM	4	9	1	8	≈5.36	≈48.23	TS-FIMM-OS	≈2.18×	≈12.13×
								TS-FIMM-P	≈3.20×	≈17.83×
								Fuzzy-PI-OS	≈1.97×	≈10.94×
								Fuzzy-PI-P	≈2.76×	≈15.37×
[14] (2018)	M-IM	2	25	1	16	≈1.67	≈41.75	TS-FIMM-OS	≈6.75×	≈13.23×
								TS-FIMM-P	≈10.17×	≈19.93×
								Fuzzy-PI-OS	≈5.74×	≈11.25×
								Fuzzy-PI-P	≈8.03×	≈15.74×
[18] (2019)	M-IM	2	25	1	8	≈1.00	≈25.00	TS-FIMM-OS	≈11.94×	≈23.40×
								TS-FIMM-P	≈17.55×	≈34.40×
								Fuzzy-PI-OS	≈10.77×	≈21.11×
								Fuzzy-PI-P	≈15.13×	≈29.65×
[20] (2019)	M-IM	3	42	1	−	≈1.00	≈42.00	TS-FIMM-OS	≈11.94×	≈13.85×
								TS-FIMM-P	≈17.55×	≈20.36×
								Fuzzy-PI-OS	≈10.77×	≈12.49×
								Fuzzy-PI-P	≈15.13×	≈17.55×
[7] (2019)	TS-IM	3	−	2	24	≈1.56	−	TS-FIMM-OS	≈7.23×	−
								TS-FIMM-P	≈10.88×	−
								Fuzzy-PI-OS	≈6.15×	−
								Fuzzy-PI-P	≈8.59×	−

**Table 8:** Hardware occupation comparison with other works.

References	FPGA	NLC	NMULT	NBitsM	This Work	$R_{\mathrm{occupation}}$		
						NLC	NMULT	NBitsM
[11] (2013)	Spartan 3A	447	4	1512K	TS-FIMM-OS	≈26.24×	≈12.25×	$\approx 10^{-6}\times$
					TS-FIMM-P	≈22.61×		
					Fuzzy-PI-OS	≈26.24×		
					Fuzzy-PI-P	≈22.61×		
[5] (2014)	Cyclone II	1622	0	8.19K	TS-FIMM-OS	≈6.51×	49×	$\approx 10^{-3}\times$
					TS-FIMM-P	≈5.51×		
					Fuzzy-PI-OS	≈6.74×		
					Fuzzy-PI-P	≈5.75×		
[16] (2015)	Arria V GX	6496	0	6.592K	TS-FIMM-OS	≈2.53×	49×	$\approx 10^{-3}\times$
					TS-FIMM-P	≈2.21×		
					Fuzzy-PI-OS	≈2.61×		
					Fuzzy-PI-P	≈2.29×		
[31] (2016)	Spartan 6	871	32	0K	TS-FIMM-OS	≈12.13×	≈1.53×	1×
					TS-FIMM-P	≈10.28×		
					Fuzzy-PI-OS	≈12.56×		
					Fuzzy-PI-P	≈10.71×		
[14] (2018)	Spartan 6	11425	5	0K	TS-FIMM-OS	≈1.44×	≈9.8×	1×
					TS-FIMM-P	≈1.25×		
					Fuzzy-PI-OS	≈1.48×		
					Fuzzy-PI-P	≈1.30×		
[20] (2019)	Virtex 5	13108	53	0K	TS-FIMM-OS	≈1.25×	≈0.93×	1×
					TS-FIMM-P	≈1.09×		
					Fuzzy-PI-OS	≈1.29×		
					Fuzzy-PI-P	≈1.13×		
[7] (2019)	Virtex 7	12468	38	0K	TS-FIMM-OS	≈1.32×	≈1.29×	1×
					TS-FIMM-P	≈1.15×		
					Fuzzy-PI-OS	≈1.36×		
					Fuzzy-PI-P	≈1.19×		

**Table 9:** Dynamic power comparison with other works.

References	FPGA	$N_{g}^{\mathrm{ref}}$	$F_{\mathrm{clk}}^{\mathrm{ref}}$ (MHz)	This Work	$N_{g}^{\mathrm{work}}$	$F_{\mathrm{clk}}^{\mathrm{work}}$ (MHz)	$S_{d}$
[11] (2013)	Spartan 3A	451	66.251	TS-FIMM-OS	11779	6.63	≈38.20×
				TS-FIMM-P	10,157		≈44.30×
				Fuzzy-PI-OS	11,779		≈38.20×
				Fuzzy-PI-P	10,157		≈44.30×
[16] (2015)	Arria V GX	6496	125	TS-FIMM-OS	16,453	0.51	$\approx 10^{6}\times$
				TS-FIMM-P	14,377		
				Fuzzy-PI-OS	17,001		
				Fuzzy-PI-P	14,926		
[31] (2016)	Spartan 6	903	20	TS-FIMM-OS	6598	5.36	≈4.42×
				TS-FIMM-P	5590		≈5.22×
				Fuzzy-PI-OS	6834		≈4.27×
				Fuzzy-PI-P	5826		≈5.01×
[14] (2018)	Spartan 6	11430	10	TS-FIMM-OS	10,252	1.67	≈149.16×
				TS-FIMM-P	8955		≈170.70×
				Fuzzy-PI-OS	10,595		≈144.35×
				Fuzzy-PI-P	9298		≈164.42×
[7] (2019)	Virtex 7	12506	150	TS-FIMM-OS	10,252	1.56	$\approx 10^{5}\times$
				TS-FIMM-P	8955		
				Fuzzy-PI-OS	10,595		
				Fuzzy-PI-P	9298		
